# Comment on “The Diagnostic Performance of the AlzoSure Predict Assay and Its Association With Alzheimer's Disease Biomarkers and Imaging Findings”

**DOI:** 10.1002/hsr2.73152

**Published:** 2026-08-30

**Authors:** Muhammad Ahmad Khan, Nida Sher

**Affiliations:** ^1^ Nowshera Medical College Nowshera Pakistan; ^2^ Khyber Girls Medical College Peshawar Pakistan

## Author Contributions

**Muhammad Ahmad Khan:** conceptualization, methodology, investigation, validation. **Nida Sher:** formal analysis, supervision, visualization, project administration. All authors have read and approved the final manuscript.

## Funding

The authors have nothing to report.

## Ethics Statement

The authors have nothing to report.

## Consent

The authors have nothing to report.

## Conflicts of Interest

The authors declare no conflicts of interest.

## Transparency Statement

The corresponding author (Muhammad Ahmad Khan) affirms that this manuscript is an honest, accurate, and transparent account of the study reported; that no important aspect of the study has been omitted.

Dear Editor,

We read with great interest the study by Rajabpour‐Sanati et al. Assessing the diagnostic performance of the AlzoSure Predict assay and its link to established Alzheimer's disease (AD) biomarkers [1]. The authors deserve praise for examining the relationship of plasma U‐p53AZ to CSF tau markers, and for recognizing the relatively poor discriminatory ability of the assay. However, two methodological issues are worth further discussion in the interpretation of the clinical implications of these findings.

First, the study did not define whether CSF total tau and phosphorylated tau181 are incremental diagnostic markers when added to existing blood‐based markers in patients with U‐p53AZ. Second, the study did not account for the potential for CSF total tau and phosphorylated tau181 to differ from each other in the way they predict TDP‐43 changes. Third, the study did not investigate the incremental diagnostic value of CSF total tau and phosphorylated tau181 in patients with normal CSF tau levels and blood‐based markers. Recent studies have demonstrated that plasma p‐tau181 and especially p‐tau217 have superb diagnostic value, strong correlation with amyloid pathology, and good prognostic value throughout the Alzheimer's disease (AD) continuum. Therefore, demonstrating statistically significant association with CSF tau does not necessarily mean that U‐p53AZ adds clinically meaningful information. Since no direct comparisons have been made with these accepted biomarkers and formal incremental predictive testing comparisons like net reclassification improvement, integrated discrimination improvement, or decision‐curve analysis have been performed, it is unclear whether AlzoSure demonstrates incremental clinical value or simply reflects the pathological process already covered by the existing plasma markers [2, 3].

Second, the diagnostic performance was reported only with respect to comparisons made between cognitively normal subjects and patients with MCI. This design is useful for the study and evaluation of early disease processes, but it is not suitable for the clinical problem of differentiating AD from other neurodegenerative dementias that often exhibit similar clinical cognitive syndromes. Ideally, biomarkers for routine clinical use should be sufficiently specific in a wide range of dementia patients, rather than just a particular point in the disease process. Thus, the diagnostic accuracy figures reported may overestimate the ability of this assay to be applicable in real‐life memory clinic contexts [4, 5].

To conclude, the results add to the emerging body of evidence that blood markers are useful in the diagnosis of Alzheimer's disease, but more research is required to compare the usefulness of U‐p53AZ with other known plasma markers and in more diverse clinical settings to establish whether it is an incremental diagnostic tool.

## Data Availability

Data sharing is not applicable to this article as no datasets were generated or analyzed during the current study. No new data were analyzed or generated in support of this research.
